# Examination of the psychometric properties of Arabic version of the Body Vigilance Scale

**DOI:** 10.1371/journal.pone.0324610

**Published:** 2025-06-02

**Authors:** Abdallah Chahine, Ali Hemade, Christian-Joseph El Zouki, Sahar Obeid, Jean-Claude Lahoud, Feten Fekih-Romdhane, Souheil Hallit

**Affiliations:** 1 School of Medicine and Medical Sciences, Holy Spirit University of Kaslik, Jounieh, Lebanon; 2 Faculty of Medicine, Lebanese University, Hadat, Lebanon; 3 UFR de Médecine, Université de Picardie Jules Verne, Amiens, France; 4 Social and Education Sciences Department, School of Arts and Sciences, Lebanese American University, Jbeil, Lebanon; 5 Department of Orthopedic Surgery and Traumatology, Notre Dame des Secours University Hospital Center, Byblos, Lebanon; 6 The Tunisian Center of Early Intervention in Psychosis, Department of Psychiatry “Ibn Omrane”, Razi Hospital, Manouba, Tunisia; 7 Tunis El Manar University, Faculty of Medicine of Tunis, Tunis, Tunisia; 8 Department of Psychology, College of Humanities, Effat University, Jeddah, Saudi Arabia; 9 Applied Science Research Center, Applied Science Private University, Amman, Jordan; University of Modena and Reggio Emilia: Universita degli Studi di Modena e Reggio Emilia, ITALY

## Abstract

**Introduction:**

The Body Vigilance Scale (BVS) was designed and validated as a short and concise measure to assess attentional focus on bodily sensations and related processes. The BVS is available in the English language, but no Arabic version have been developed, and no validation of the scale exists in Lebanon. The current study aimed to determine the reliability, validity and factor structure of the Arabic version of the Body Vigilance Scale.

**Methods:**

This study has a cross-sectional design. It was conducted from October 2 to November 20, 2024, enrolling Lebanese adults. The study was carried out in the Arabic language and included the BVS, the Patient Health Questionnaire, the Insomnia Severity Index and the Freiburg Mindfulness Inventory.

**Results:**

In total, 641 participants participated in this study, with a mean age of 35.11 ± 12.67 years and 70.5% females. Internal reliability of BVS was adequate (ω = .87/ α = .86). Invariance was shown at the metric and scalar levels in terms of genders. A significantly higher mean BVS score was found in females compared to males. Higher depression (r = 0.26; p < 0.001), anxiety (r = 0.29; p < 0.001), insomnia (r = 0.29; p < 0.001) and mindfulness (r = 0.27; p < 0.001) correlated significantly with higher body vigilance scores.

**Conclusion:**

The Arabic version of the BVS is a reliable and valid tool for assessing somatic attention in Arabic-speaking populations. Its psychometric robustness, demonstrated measurement invariance across genders, and associations with psychological distress measures underscore its utility in both clinical and research settings.

## Introduction

Bodily sensations are defined as the translation of feelings or sensory experiences into body symptoms like fatigue, aches, pain, or even pleasure [1]. This phenomenon, which is a cross-link between psychological and physical processes, is called body vigilance, or when someone is aware of their body and pays attention to internal sensations [2]. Body vigilance is considered a normal adaptive process to external and internal factors that may affect a person’s life. It involves the interpretation of sensations as a threat or a sign of alarm, the hypervigilance, which is the constant checking for physical symptoms, and the interference with daily life, which describes how much body vigilance distorts the normal functioning of the person in question [3]. Several components enter in shaping body vigilance, from selective focus to certain symptoms to increased attention and false linking of bodily sensation and hypervigilance [4]. One of the most important components is the frequency of attention to bodily sensations, which describes how much someone pays attention to his internal bodily sensations in situations of deviation from homeostasis [4]. Also, another crucial component of Body Vigilance is the perceived intensity of sensations, which translates to how sensitive a person is to the changes he is living with.

The five major senses (sight, taste, smell, hearing, and touch) require at least one of the four basic sensory abilities (photoreception, mechanoreception, chemoreception, and thermoreception) [5], which are considered the peripheral sensory receptors and can be sensitized by stress and anxiety, making people more prone to receiving stimuli [6]. In a certain situation of homeostasis dysregulation, like a stressful event or a danger encounter, the body’s natural response (the “fight or flight” phenomenon) is driven by the sympathetic nervous system, including increasing heart rate, breathing, and increased muscle tone, among other symptoms [7]. A deviation from the norm is interpreted by someone with normal interpretation processes as a normal bodily reaction to stress, whereas hypervigilant people interpret them as a warning sign, and this can be explained by physiological mechanisms [8]. Essentially, body vigilance involves the activation and suppression of neural and hormonal pathways, i.e., interoceptive networks [8]. The autonomic nervous system (triggered by anxiety and stress) is heavily involved, whereas the parasympathetic nervous system (known for providing relaxation of the body) is downregulated and suppressed [9]. Moreover, the hypothalamic-pituitary-adrenal axis is activated in stress-induced body vigilance, specifically cortisol, the stress hormone, leading to enhancement of sensitivity to internal sensations [10]. That is why body vigilance is different from interoceptive accuracy and may more accurately reflect interoceptive sensibility, which is subjective, as these sensations and disturbances might not be reflective of true physiological processes.

Body vigilance was found to be associated with panic disorders, hypochondriasis, pain, and other somatic disorders [11]. Perhaps the strongest and most known correlation between Body vigilance and another disorder is anxiety and panic disorders [12]. Anxious individuals frequently see physical symptoms every day, such as elevated heart rate and dyspnea, as warning indications of danger, which feeds the vicious cycle of fear and hypervigilance [13]. On the other hand, Body vigilance is indirectly linked to depression [14]. Depression can lead to somatic preoccupation a condition where individual exaggerate certain symptoms and associate them with serious medical problems. They believe, for example, that a common headache is a sign of brain tumor, or chest pain is a sign of a heart attack [15]. Therefore, the sense of overload and distress in depressed individuals is explained by this negative cognitive bias [16]. Another disorder that is connected to hypervigilance is insomnia [17]. A study conducted about the effects of chronic insomnia on vigilance showed that people suffering from insomnia had a faster response to simple vigilance tasks but a much slower response to complex vigilance tasks in comparison with controls; also, sleep therapy showed an effective correction of the difference in response, showing the role played by insomnia in vigilance [18]. Another important variable that interacts with Body vigilance is mindfulness. In a study conducted in 2022, Perez-Pena et al. argued that mindfulness-based interventions have an important effect on body awareness symptomatology, including self-discrepancy and rumination, among others, by reducing them [19]. This shows that body vigilance can interact with multiple variables like anxiety, depression, insomnia, and mindfulness.

Multiple scales can be used for the measurement of bodily sensations, like the Body Awareness Questionnaire (BAQ) [20], the Somatosensory Amplification Scale (SSAS) [21], the Interoceptive Awareness Scales (IAS) [22] and the Body Vigilance Scale (BVS), but each score focuses on a slightly different aspect of body vigilance. Regarding this common and relatively new description of bodily sensations, Schmitt et al. elaborated the BVS, a widely used four-item scale that asks respondents to rate their agreement with a specific statement related to attentional focus on bodily sensations and related processes [4]. The scale is based on an eleven-point Likert-type scale that evaluates three main aspects: awareness of vigilance tendencies, monitoring, and emotional response to bodily sensations. A fourth item includes a rating of how much someone pays attention to 15 symptoms, including chest pain, tingling, numbness, shortness of breath, fainting, changes in vision, feeling unreal and detached from oneself, dizziness, hot flashes, sweating, nausea, stomach upset, and choking [4]. A high score is often indicative of hypervigilance and is often linked with anxiety and stress, and a low score is suggestive of a relatively normal bodily sensation. The BVS is considered superior to other more broad tools like the BAQ and the IAS because it centers on pathological hypervigilance and has a specific utility for anxiety and stress disorders. Adding to that, the emotional impact of body vigilance is assessed using this scale, privileging it over the others [20,23]. Also, this scale is concise and clear with a time advantage over other scales, having strong internal consistency and validity, which ensures that it correctly grasps the body vigilance of a person.

The BVS is available in the English language and has been translated to Spanish [24] and Japanese [25]. The original scale in English has been validated in clinical and non-clinical populations [26]. Imagined by Schmidt, Lerew, and Trakowski in 1997, this scale demonstrated a unidimensional factor structure, measuring only body vigilance. It also showed an adequate internal consistency with a Cronbach’s alpha coefficient of 0.75 [4]. This was confirmed by the Spanish version, supporting its one-dimensional structure and internal consistency with a Cronbach’s alpha coefficient of 0.985 [24]. Moreover, the scale presents good validity, since it is correlated significantly with measures of anxiety and panic-related symptoms, validated again by the Spanish version of the scale. No Arabic version has been developed addressing the BVS, and no validation of the scale exists in Lebanon, which raises concerns for language barriers, making it difficult to measure psychological dimensions effectively in Arabic-speaking communities.

It is well known that experiencing anxiety and stress can vary between cultures [27]. Some cultures are more prone to expressing stress emotionally, and others translate these feelings into physical symptoms [28]. Health-related symptoms are frequently manifested physically rather than emotionally in Arab societies [29]. Arabic people suffering from anxiety and panic disorders tend to describe somatic symptoms such as headaches and chest pain rather than describe emotional distress [30]. This cultural orientation may result in an increased focus on physical sensations, which is why a culturally appropriate and validated instrument such as the BVS is essential for precise evaluation in this region of the world. Additionally, Arab people are becoming more aware of mental health issues such as anxiety and stress, but underreporting and stigma frequently result in incorrect diagnoses or delayed treatment [31]. By validating BVS for the Arabic language, clinicians will be provided with a tool that is trustworthy to evaluate body vigilance, particularly in relation to anxiety disorders, enhancing diagnosis and treatment.

In Lebanon, a Middle eastern Arabic speaking country tormented by war, economic collapse, constant socio-political conflicts, and regional instability, it can be noted that anxiety, stress, and somatization are at the highest levels [32–34]. Lebanon thus serves as a place where more bodily perceptions can flourish than any other place. Therefore, our study evaluates the psychometric properties of this scale in the general population of Lebanon. The current study aimed to determine the reliability, validity, and factor structure of the Arabic version of the BVS. We hypothesize that the Arabic version of the BVS will have a structure that is unidimensional in nature. Also, we hypothesize that BVS will positively correlate with anxiety, depression, insomnia, and mindfulness.

## Methods

### Ethics approval

This study was conducted with the ethical standards specified by the Ethics Committee of Notre Dame des Secours University Hospital. Every subject was fully informed about the reason behind the research, their rights, the confidentiality of their data, and the voluntary basis of participation. Prior to completing the questionnaire, written informed consent was obtained from all participants.

### Study design and participants

This study has a cross-sectional design. It was conducted from October 2 to November 20, 2024, enrolling Lebanese adults from all over the country. A 15-minute online survey link was forwarded through the snowball method using social media and messenger apps. Participants above the age of 18, residents of Lebanon, and those who would approve of joining the study were included.

### Questionnaire

This survey was carried out in the Arabic language, the official language of Lebanon. It was divided into several sections: the first section contained the intention of the study and the electronic consent form before the start of the study. The second section outlined the general information on sociodemographics, including age, sex, and financial stress. The third is health-focused and covers questions on BMI and history of medical and surgical conditions. Smoker status was evaluated using pack years, and alcohol consumption was assessed based on questions about the number of drinks one usually consumes per week. The last section included scales and indexes as cited below.

### Body Vigilance Scale (BVS)

It is a widely used four-item scale that asks respondents to rate their agreement with a specific statement related to attentional focus on bodily sensations and related processes. The scale is based on an eleven-point Likert-type scale that evaluates three main aspects: awareness of vigilance tendencies, monitoring and emotional response of bodily sensations [4]. The translation process was done through forward and backward translation by two independent bilingual translators. A first translator transformed the text from the English language to the Arabic language and second translator conducted the translation from Arabic to English. There was no contact between translator number 1 and 2 in respect to the blinding process. The final English version was compared to the original scale and minor linguistic modifications were done. By doing this step, the original items’ meaning was preserved in the translated version. No expert panel was involved in the reconciliation process. Nonetheless, the Arabic translation was carefully edited to preserve conceptual clarity. No significant cultural adaptations were deemed necessary since the original items were relevant to Arabic-speaking populations.

### Patient Health Questionnaire (PHQ-4)

This brief scale is composed of 4 items that are used to evaluate symptoms of depression and anxiety throughout the previous two weeks [35]. It consists of two sub-scales: Depression and Anxiety. It consists of a 4-point Likert scale, with 0 denoting “not at all” and 3 denoting “almost every day.” The addition of the scores for each of the four PHQ-4 items determines the final score. A score of three and above indicates mild psychological suffering, six to eight indicates moderate psychological distress, and nine to twelve indicates severe psychological distress. The validated Arabic version was used in this study [36].

### Insomnia Severity Index (ISI)

It comprises a self-administered, widely used 7-item questionnaire that focuses on the DSM IV diagnostic criteria for insomnia, particularly created to assess how patients perceive insomnia and how it affects their quality of life, ability to operate daily and to maintain their sleep, and their level of concern about their sleeping problems [37].

### Freiburg Mindfulness Inventory (FMI)

This is a reliable scale that is composed of 14 items covering all aspects of mindfulness. It can be scored as 1 for “Rarely”, 2 for “Occasionally”, 3 for “Fairly often” and 4 for “Almost always” according to the participant’s own experience [38]. The Arabic version was used in this study, with higher scores reflecting more mindfulness [39] [ref].

### Analytic strategy

We conducted a CFA via the SPSS AMOS v.28 software. We estimated a minimum sample of 80 participants based on the recommendation of 20 times per scale’s variables [40]. The maximum likelihood method was used to obtain parameters estimate. Multiple fit indices were calculated: root mean square error of approximation (RMSEA) (≤0.08), standardized root mean square residual (SRMR) (≤0.05), Tucker-Lewis Index (TLI) and Comparative Fit Index (CFI) (≥.90 for both) [41]. Additionally, convergent validity was checked via the average variance extracted (AVE) ≥.50 [42]. Multivariate normality was not verified at first (Bollen-Stine bootstrap p = 0.002); therefore, we performed non-parametric bootstrapping procedure.

A multi-group CFA was conducted to examine measurement invariance of BVS scores between genders [43] at the configural, metric, and scalar levels [44]. ΔCFI ≤ .010 and ΔRMSEA ≤ .015 or ΔSRMR ≤ .010 supported the evidence of invariance [45]. Comparison of BVS scores between genders was done using the student’s t test.

Composite reliability was assessed using McDonald’s ω and Cronbach’s α, with values greater than 0.70 reflecting adequate composite reliability [46]. The BVS scores were considered normally distributed as shown by skewness and kurtosis values between -1 and +1 [47]. The association between the ANLS scores and other scores was evaluated using the Pearson test.

## Results

In total, 641 participants participated in this study, with a mean age of 35.11 ± 12.67 years and 70.5% females. Other descriptive statistics of the sample can be found in Table 1.

**Table 1:** Sociodemographic and other characteristics of the sample (N = 641).

Variable	N (%)
Sex	
Male	189 (29.5%)
Female	452 (70.5%)
Education level	
Secondary or less	124 (19.3%)
University	517 (80.7%)
Marital status	
Single	355 (55.4%)
Married	286 (44.6%)
	**Mean **± **SD**
Age (years)	35.11 ± 12.67
Household crowding index (person/room)	1.14 ± 0.97
Body Mass Index (kg/m^2^)	25.29 ± 7.36
Depression	2.40 ± 1.94
Anxiety	2.31 ± 2.05
Insomnia	11.22 ± 5.96

### Confirmatory factor analysis

**Fig 1:**
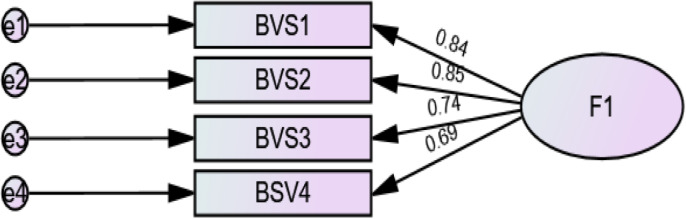
Standardized loading factors deriving from the confirmatory factor analysis of the Body Vigilance Scale.

The fit indices were good (RMSEA = 0.118 (90% CI 0.075, 0.168), SRMR = 0.025, CFI = 0.985, TLI = 0.956). The standardised estimates of factor loadings were all adequate (Fig 1). Internal reliability was adequate (ω = .87/ α = .86).

### Gender invariance

Invariance was shown at the metric and scalar levels in terms of genders (Table 2). A significantly higher mean BVS score was found in females compared to males (17.69 ± 9.27 vs 17.69 ± 10.81; *p* = 0.002, Cohen’s d = 0.255).

**Table 2:** Measurement invariance of the Body Vigilance Scale across sex.

Model	CFI	RMSEA	SRMR	Model Comparison	ΔCFI	ΔRMSEA	ΔSRMR
Configural	0.985	0.083	0.025				
Metric	0.986	0.060	0.023	Configural vs metric	0.001	0.023	0.002
Scalar	0.985	0.053	0.023	Metric vs scalar	0.001	0007	<0.001

CFI = Comparative fit index; RMSEA = root mean square error of approximation; SRMR = Standardised root mean square residual.

### Concurrent validity

Higher depression (r = 0.26; p < 0.001), anxiety (r = 0.29; p < 0.001), insomnia (r = 0.29; p < 0.001) and mindfulness (r = 0.27; p < 0.001) correlated significantly with higher body vigilance scores.

## Discussion

The present study aimed to validate the psychometric properties of the Arabic version of BVS among a sample of Arabic-speaking adults. The results provide compelling evidence for the reliability, validity, and cross-gender applicability of the scale, contributing to the existing literature on the assessment of somatic attention and awareness in diverse populations.

The CFA confirmed the factor structure of the Arabic BVS, with fit indices (– RMSEA = 0.118, CFI = 0.985, and TLI = 0.956) demonstrating acceptable model fit. These findings align with prior validations of the BVS across different cultural contexts, where similar goodness-of-fit indices were reported [24–26]. In comparison with other validations of the BVS, all versions showed a unidimensional structure. Our version focuses on the general population while the Spanish version worked mainly on and the Japanese version focused on, but it is worth noting that the English version of the scale, which is the original version, was validated for clinical and non-clinical populations. Also, the other versions showed good results. The Spanish version of the scale showed an excellent internal reliability with a Cronbach’s alpha of 0.985 in subjects affected by panic disorder as a self-reported diagnosis, and the Japanese version showed modest results with a Cronbach’s alpha of 0. 79 in university students [24,25]. The strong internal reliability of the Arabic version (McDonald’s ω = 0.87 and Cronbach’s α = 0.86) further underscores the robustness of the scale. It is worth noting that a study conducted by Bernstein et al., about the cross-cultural validation of the BVS in non-clinical populations in English and Spanish showed poor fit for the four-items model but good fit for the three-items, unidirectional model [2] which is different from our results that showed good fit with the four-items model. These differences can be explained by cultural differences.

While the CFA of the BVS demonstrated good fit across most indices, the RMSEA value (0.118) exceeded conventional acceptability thresholds. This implies a certain level of model misfit, which could be explained by the factor structure of the scale. Future research is needed to explore a reduced-item model to reevaluate the factor structure and if a fewer items model can offer a more reliable assessment than the original version.

Measurement invariance analysis revealed that the scale performs equivalently across genders at both metric and scalar levels. This is a critical finding, as it confirms the absence of gender-related bias in the Arabic BVS, ensuring its utility for comparative studies. Gender differences were observed, with females reporting higher BVS scores than males. This result aligns with evidence suggesting heightened interoceptive awareness in females due to sociocultural and biological factors, such as greater sensitivity to internal bodily cues and societal pressures to monitor one’s physical state [48]. Heightened BVS scores in females may also reflect societal expectations for women to maintain a greater focus on physical appearance and health, contributing to higher levels of body vigilance [49]. Biologically, research indicates that hormonal variations and brain regions associated with emotion and interoception, such as the insula, are more active in females, potentially leading to increased attention to bodily sensations [50,51]. These findings collectively highlight the interplay of biological and sociocultural factors in explaining gender differences in body vigilance.

The significant correlations between BVS scores and measures of depression, anxiety, and insomnia provide strong evidence for the concurrent validity of the scale. These findings echo prior studies linking heightened body vigilance to increased psychological distress, suggesting that excessive focus on bodily sensations is associated with symptoms of anxiety and somatic complaints [14,52]. Notably, the positive association with mindfulness highlights a dual aspect of body vigilance, wherein heightened awareness of bodily sensations may either contribute to distress or facilitate adaptive outcomes, depending on contextual factors and individual differences. This interesting positive correlation can be explained by the difference between adaptive body awareness and maladaptive hypervigilance. Mindfulness promotes nonjudgmental, present-moment awareness, which may improve interoceptive sensitivity in a more adaptive manner than hypervigilance, which includes an intense, frequently anxiety-driven focus on physiological sensations. As a result, those with high mindfulness scores might be more aware of their bodies without necessarily feeling the anxiety that comes with hypervigilance [53,54].

### Limitations

The present study, while offering valuable insights into the psychometric properties of the Arabic version of the BVS, is not without limitations. First, the sample predominantly consisted of young adults, with a higher representation of females, potentially limiting the generalizability of findings to older populations or more gender-balanced samples. With 70% of the sample being of the female gender, results should be interpreted with caution because of the risk of overrepresentation as previous research showed that body vigilance and interoceptive awareness can differ by gender, with females typically reporting higher levels. Besides, most of our participants had a university-level education (80.7%), suggesting that the sample may not fully represent lower socio-economic status individuals. Second, the reliance on self-reported measures introduces the possibility of response biases, such as social desirability or misinterpretation of scale items, which could affect the accuracy of the results. Third, the study employed a cross-sectional design, which restricts the ability to draw causal inferences about the relationships between body vigilance and psychological outcomes like anxiety, depression, and insomnia. Fourth, while the study successfully established measurement invariance across genders, other potential moderators, such as socioeconomic status or cultural variations within Arabic-speaking populations, were not explored, which may influence the applicability of the findings in diverse subgroups. Lastly, the study did not examine the predictive validity of the BVS or its sensitivity to change over time, which are crucial for understanding its utility in longitudinal research or intervention contexts. Addressing these limitations in future research will enhance the scale’s robustness and applicability in varied contexts.

### Implications and future directions

The validated Arabic BVS has significant implications for both clinical practice and research. Clinically, the scale can be employed as a diagnostic and screening tool to assess somatic attention in Arabic-speaking populations, particularly in those presenting with anxiety, somatization, or related disorders. This tool may also be valuable for evaluating the efficacy of interventions aimed at reducing body vigilance, such as mindfulness-based therapies.

From a research perspective, the BVS provides a standardized measure for cross-cultural studies exploring the role of body vigilance in mental health outcomes. The demonstration of measurement invariance across genders ensures its utility in comparative studies, facilitating nuanced explorations of how sociocultural and biological factors influence somatic awareness.

Future research should aim to validate the Arabic BVS in diverse subpopulations, including clinical groups and older adults, to enhance its applicability. Longitudinal studies are needed to investigate the causal relationships between body vigilance and mental health outcomes. Additionally, exploring the role of cultural factors in shaping body vigilance could provide deeper insights into its variability across different contexts. Finally, intervention studies assessing the impact of mindfulness and body-focused therapies on BVS scores would be valuable. We should note that due to the limitations of our study and the study design, results should be interpreted with caution and cannot be generalized. More longitudinal research should be done to establish a causative relationship. Also, research with a more representative sample is needed to better represent the Lebanese population.

## Conclusion

The Arabic version of the BVS is a reliable and valid tool for assessing somatic attention in Arabic-speaking populations. Its psychometric robustness demonstrated measurement invariance across genders, and associations with psychological distress measures underscore its utility in both clinical and research settings. This study contributes to the growing literature on culturally adapted psychometric tools, paving the way for more inclusive research and clinical practice.
